# Hospital and Institutional News

**Published:** 1915-05-15

**Authors:** 


### HOSPITAL AND INSTITUTIONAL NEWS.

### THE BUSINESS OF WAR.

THE British nation is suffering at the moment from a failure to recognise that war to be successful must be conducted as a business on business principles. In view of the German methods of barbarism and their setting aside of all the safeguards instituted by civilised nations to govern the conduct of war and for the protection of neutral countries and innocent and defenceless men, women, and children, it is essential that everybody in the British Empire, from members of the Government to the humblest trader, shall put the security of British interests before all other considerations. We hold that every British newspaper should decline to give publicity to the advertisements and other copy supplied by Germans and German-owned enterprises. All British newspapers of influence and repute must refuse to insert any advertisements of any kind emanating from these sources. In pursuance of this policy we have this week felt it our duty to reject whole-page advertisements of the kind referred to.

### WOUNDED SOLDIERS AT VOLUNTARY HOSPITALS.

THERE is not a voluntary hospital in the United Kingdom which has not gladly placed at the disposal of the military authorities every available accommodation and all possible means which may in any way contribute to the comfort and speedy recovery of the wounded. At the commencement of the year a careful calculation, based upon the available facts, indicated that if the war continued the additional cost of this extra work to the voluntary hospitals during 1915 would approach, if it did not exceed, half a million pounds. Not only has the war continued but it has gravely increased in violence, and its effects upon the fighting men have produced results which render it essential that at least double the hospital provision available in January 1915 shall be forthcoming by the end of the present month. It is clear, therefore, that the voluntary hospitals must require during 1915 an additional income of at least one million pounds over and above their average income during the previous three years. These facts from the financial point of view are serious, but if they are faced in the right spirit and the newest methods of attracting money are utilised by every voluntary hospital throughout the land, we shall have no anxiety for the result.

### THE NORWICH £5 PER BED SCHEME.

NORFOLK has had more than its share of attention from German aircraft and German guns. This attention on the part of the enemy has stiffened the backbone of the inhabitants of East Anglia and united them as one man and one woman, to say nothing of the children. Everybody from the highest to the lowest is alive and awake and all are doing their best to lend a useful hand. A scheme for the maintenance of the beds occupied by soldiers at the Norfolk and Norwich Hospital has attained remarkable popularity. Two hundred and five beds are at present occupied by soldiers from the Expeditionary Force, and this number is likely to be increased to 250. Since the middle of October 1,890 soldiers have been admitted. It would be interesting if every hospital secretary would send us a postcard giving similar figures for his institution covering the same period. Finding that the allowance granted by the Government was quite inadequate, a scheme for the maintenance of soldier beds was suggested which enabled any firm, or society, or individual to contribute £5 per bed per month towards the cost of its maintenance. This plan has received its chief support from the employees in various factories. In smaller establishments the proprietors and directors have joined with the employees to collectively contribute at least £5 a month towards one bed. Individuals have asked for the privilege of sending £5 a month, and the same course has been adopted by domestic servants in families, teachers, various associations, villages, and parishes throughout the county, the wards of the city of Norwich, a few friends in unison, and residents in streets, roads, and squares. Splendid is the word used by our correspondent to describe the results achieved by this scheme, which has taken the public fancy and has already resulted in a provision for the maintenance of 118 out of the 205 beds allocated to soldiers.

### THE MONETARY VALUE OF EXAMPLE.

THE £5 a bed scheme at the Norfolk and Norwich Hospital in attaining its immediate object has proved otherwise fruitful in the provision of money and means. The scheme possessed the attraction of novelty and was given a direct personal touch by the Board's agreeing to the admission of two visitors to each £5 bed on Sundays between 3 and 4 P.M. This privilege has kept the interest alive. The people are able to satisfy themselves that their subscriptions are being applied to a real object; they leave the hospital full of enthusiasm, and by spreading abroad the news of what they have seen have secured an ever-increasing number of citizens as contributors. A further effect has been to cause a considerable sum to flow in each month from all directions to the general funds. Again, those unable to send money exert themselves in various ways, and 15,000 eggs were thus collected and delivered at the Norfolk and Norwich Hospital during the month of April, in addition to innumerable other gifts in kind. The county of Norfolk is thoroughly alive and awake. It is at present providing for the maintenance of 37 Voluntary Aid Detachment hospitals, to which the wounded are transferred from the Norwich Hospital to complete their convalescence, under the direction of the honorary medical staff. In our Report on the Norfolk and Norwich Hospital, published January 13, 1914, we ventured to forecast a rapid development in activity at this hospital. It is far removed from the centre, but the people and their hospital are unusually inter-dependent, afact which adds to the credit and value of the new scheme that has proved so successful at Norwich. This £5 per bed plan is one excellent outcome of the Great War. It well illustrates how its demands upon every individual resident throughout the British Islands can be readily and effectively met.

### BRISTOL'S NEW WAR HOSPITAL.

At a meeting of the Bristol Branch Executive Committee of the British Red Cross Society held on Thursday, April 29, it was stated that the Bristol Lunatic Asylum has been secured for use as a military hospital, and that it will be ready for occupation almost immediately. The institution, which is capable of accommodating some 1,040 patients, will be known as the Beaufort War Hospital.

### INCOME TAX AND HOSPITAL SALARIES.

The question whether hospital authorities should pay income tax upon the salaries of their officers has been lately raised, and the recent decision of the governors of St. Bartholomew's Hospital is worth recording. The governors have agreed, having regard to "the obligation" created by an order of the Court in 1846, to continue to pay the income tax upon the salaries of the officers at present in the service. The rule is not, however, to apply to officers who may be appointed in the future.

### A MATERNITY CENTRE IN THE POTTERIES.

The Borough Council of Stoke-on-Trent has resolved on the establishment of a maternity centre with branches at Longton, Etruria, and Burslem. The scheme provides for the care of expectant mothers, advice at clinics, and the provision of skilled attendance during childbirth. In addition, lady visitors will be appointed who will be ready to give advice and help in the rearing of children. The annual cost of this programme will be £2,300, and half of the charges will be met by a grant from the Local Government Board.

### TYPHUS IN SERBIA.

Professor Morrison, of Birmingham, who has recently returned from Serbia, is able to make the comparatively satisfactory statement that the ravages of typhus are abating and that the necessary medical arrangements are gradually being perfected all over the country. Some indication of the terrible character of the epidemic can be gathered from the statement that of the 300 doctors who formed the military medical staff 120 have died, the majority from typhus fever. Several volunteer doctors from other nationalities have, too, fallen victims to the disease, and the nursing staffs have not escaped.

### INVALIDS AT THE FRONT.

There is no doubt that a certain number of men have managed to pass the doctor and enter the Army in spite of the fact that they are suffering from organic disease; and some of these have apparently been none the worse for the experience. Men with phthisis pulmonalis and others with cardiac valvular disease have indeed rendered excellent service and have kept fit and strong. But to suggest from this experience, as some correspondents seem to do, that all persons so afflicted might, if they so wish, be allowed to enter the Army is really too ridiculous. There are degrees of disease to be considered, and exceptional instances are a poor basis for a general rule.

### WAR CHANGES AT THE EPILEPTIC COLONY.

The services of Dr. Petrie, assistant medical officer at the Epileptic Colony, have been transferred temporarily to Claybury Mental Hospital, to take the place of the second assistant medical officer there, who has been permitted to accept a commission in the Royal Army Medical Corps. An arrangement has been made for medical assistance to be provided for the colony from time to time from a neighbouring mental hospital, and for the dispensing to be done daily by the dispensing chemist of the same hospital.

### PUBLIC HEALTH IN PARIS.

The conditions in Paris at present are decidedly abnormal, yet it is of interest to note the municipal statistics of the city. According to the latest returns the number of deaths in the previous week was 997, as compared with 1,014, the average for the corresponding week of the last five years. Measles accounted for forty-two deaths, pneumonia for thirty-five, and influenza for ten. The figures in these and other respects show an improvement on those of the preceding week. The births registered were 892—that is 16.2 per 1,000 of the inhabitants. The average of the last five years only gives the small figure of 18 per 1,000.

### SUCCESS OF A WOMAN PHARMACIST.

The Pereira Medal, the highest award available for pharmaceutical students, has this year been won by Miss Dora F. White. This is the second time a woman pharmacist has achieved this honour, a previous winner being Miss Gertrude H. Wren, to whom the medal was awarded in 1908. The medal was struck in memory of the late Jonathan Pereira, whose works on *materia medica* are classics, and has been competed for each year for more than half a century. It is only in comparatively recent years that the woman pharmacist has become a conspicuous figure, and the fact that the medal has twice been awarded to women pharmacists in seven years is significant. The winner of the Pharmaceutical Society's silver medal this year is Mr. Arthur J. Somer, and of the bronze medal Mr. R. W. Bowles.

### CUSTOM AND HABIT.

Some of the younger governors of St. Bartholomew's Hospital rubbed their eyes when they received a notice "inviting their attendance at the Annual View of the patients in the hospital," to be held at half-past two o'clock in the afternoon of May 12. The invitation suggests many difficulties and not a few problems. These, Mr. Thomas Hayes, with his usual ability, no doubt dispersed, when the expectant governors assembled in the Great Hall "to view the patients" on the anniversary of Florence Nightingale's birthday. We wonder whether the patients were allowed to have visitors at 2.30 on the same afternoon. If they were, it is possible that the humorous amongst them may have sent to some of last year's patients, "inviting their attendance at the Annual View of the governors in the hospital on May 12, 1915."

### A SHORT WAY WITH DEFECTIVES.

In a leading article dealing with the defects discovered among school children a contemporary journal is not wanting in strenuousness. It claims that our knowledge of heredity is so confident that the occurrence of such defects may be foreseen, and, if only vigour is applied, may, too, be prevented. Prevention is the friend, not cure, and the short way is to prevent certain persons acquiring the dignity of parenthood. The argument is frank to a fault—"When our farmers deal with their flocks and herds they take every precaution that the breed should be pure, yet so overrun is this country with snobs, faddists, and ignoramuses that the purity and physical stability of humanity is left to chance." Poor country! but perhaps we may hope that this triple row of obstructives is now finding useful occupation at the Front.

### RADIUM AT MANCHESTER INFIRMARY.

Some particulars of the Radium Department at the Manchester Royal Infirmary have been given to the local Press, and it is announced that so active has been the department that the infirmary committee has found it necessary to purchase another £5,000 worth of radium. The particulars and figures of the report do not make any sensible addition to what has already been announced from similar organisations elsewhere. But it is due to local interests and support that a general account of the working of the Department should be published, and independent investigations which tend to check and qualify one another have always proved useful agencies in the search for scientific truth and stimulating influences in the direction of practical progress.

### HAMPSTEAD GENERAL HOSPITAL.

In view of the feeling which prevailed in hospital circles on the occasion of the appointment, a few months ago, of a secretary to the Hampstead General Hospital in place of the late Sergeant A. E. Thomas, it will be learnt with interest that Mr. J. A. Kyle, who was then appointed, has resigned. We understand that Mr. Kyle has enlisted in the Transport Division of the Army Service Corps. He has been in office at Hampstead for barely four months. In that time Mr. Kyle has given proof of his ability, and his resignation is a loss to hospital workers. It may be noted that the advertisement of the vacant secretaryship now appearing does not stipulate for previous hospital experience on the part of candidates, and therein differs from former advertisements. It was the appointment of Mr. Kyle in face of that stipulation which created criticism in hospital circles and gave dissatisfaction to some hospital officers.

### ABERDEEN DOCTORS AND THE WAR.

The patriotism of the North is proverbial, and the Highlands and Islands have contributed largely to the fighting ranks. On the medical side there is a similar devotion to the national cause, and there has been a wide response to the War Office appeal. A number of Aberdeen practitioners have given up their practices and have accepted commissions, and they are able to count on the loyal co-operation of those they leave behind for the protection of their interests. It is calculated that their departure means a necessity for the provision of medical attendance for something like 10,000 patients. To meet this position a central bureau and consulting rooms have been arranged, and a staff of doctors has undertaken to accept these patients until their usual medical advisers return to civil duty. The example is an excellent one, and might well be followed in other places. In saying this we do not forget that arrangements having a similar purpose have been organised in several other towns both in England and in Scotland.

### OPEN-AIR WORK FOR TUBERCULOUS PATIENTS.

From many public bodies charged with the care of tuberculous patients come complaints that the benefit derived from sanatorium treatment is very often promptly lost when the patient returns to his former surroundings; and for this and other reasons there seems to be a falling enthusiasm for the crusade against tuberculosis. A praiseworthy attempt to meet the difficulty just referred to has been made in Birmingham, where the City Council was urged to permit the use of certain selected areas in the public parks as places in which men who had been under sanatorium treatment could work as gardeners. The Council felt unable to assent to this suggestion, but they agreed to employ a limited number of men at the Quinton nurseries, and to permit the Insurance Committee to erect a chalet on the nursery ground. This seems a very sensible proceeding, and it will be watched with interest. The tuberculosis crusade has lost something of its first vigour, but, with such modification as experience suggests, it is of the highest importance to the national health.

### THE WORK AT THE ROYAL EYE HOSPITAL.

The Royal Eye Hospital at St. George's Circus, Southwark, like many other institutions dependent on voluntary subscriptions, has felt the effects of the war, and the appeals made for the various agencies connected with medical and nursing requirements of the Forces. Last year the receipts were £1,500 less than the expenditure, although since the opening of the hospital in 1857 the patients have increased from 298 to 722 in-patients and over 20,000 out-patients. At the outbreak of the war the Council felt it to be their duty to offer two wards for the treatment of wounded soldiers and sailors, and to meet their special needs an *x*-ray apparatus has been installed at a cost of £300, by which the staff are enabled accurately to localise fragments of shell and bullets. Another innovation is the appointment of a woman house surgeon, Dr. Brander. In order to continue the good work, not only among the wounded soldiers and sailors, but among the poor of South London, it is urged that, as the Royal Eye Hospital is the only one south of the Thames dealing specially with the eye, it should be placed on a basis free from financial troubles.

### THE INSURANCE ACT IN PEMBROKESHIRE.

At the recent meeting of the Pembrokeshire Insurance Committee the financial position was admitted to be anything but a cheerful one. The doctors, it was stated, were to be paid 80 per cent. of the amount due to them, and the chemists only 35 per cent. For the rest, the Committee encouraged the hope that the ensuing quarter would bring less sickness and more money. One of the members complained bitterly of the administration of the Act, and doubted whether even its author thoroughly understood it. He criticised adversely the regulation restricting the hours within which, save in exceptional circumstances, insured persons can summon the doctor, and he looked back not without regret to the old days of the clubs and the friendly societies. Under existing conditions, however true all this may be, we can only recommend the critical councillor to make the best of circumstances.

### THE SHORTAGE OF DOCTORS.

Evidence of the diminished number of doctors available for the needs of civil practice is beginning to be noted in the accounts of inquests conducted in various parts of the country. During the last few weeks several examples have occurred in which the services of a doctor could not be obtained. At one of these a practitioner, who had been sent for, explained that the pressure of work upon him had been so great that he had been quite unable to attend. On another occasion, in somewhat similar circumstances, the Coroner ventured the remark that if a doctor could not attend when summoned he ought either to keep an assistant or provide a substitute. The *obiter dicta* of judicial persons, even of those high in the legal hierarchy, are not always shining examples of wisdom, and judges of summary jurisdiction are in this respect even less successful. The chains of authority have made their weight felt in recent years, but surely we have not come to such a pass that we must keep assistants if a Coroner so decrees. Offices may be ancient without being venerable.

### PREMATURE DISCHARGES FROM OPHTHALMIC SCHOOLS.

The Metropolitan Asylums Board have found that at their ophthalmic schools several cases have recently occurred in which children under treatment have been discharged by order of the Guardians, presumably at the request of the parents, before being certified as cured by the ophthalmic surgeon. It has also been observed that during the past few months there has been a marked falling off in the number of cases admitted. The suggestion has been made that possibly one of the causes contributing to these results is the extra allowance granted by the War Office to the wives of men serving with the Forces in respect of dependent children. Attention was called to a special case which had occurred in Southwark, where a lad suffering from acute trachoma was discharged by order of the Guardians though not certified by the surgeon as cured. The Southwark Board of Guardians instituted inquiries into the case, and elicited the very strange fact that the lad in question had since his discharge from the school obtained a situation as "a doctor's boy." This fact, the Guardians held, rendered them powerless to move further in the matter. Who is the "doctor"?

### SOME SCOTTISH MEDICAL APPOINTMENTS.

The English Board of Education, in view of the medical demands of the war, decided some little time ago to authorise in various districts some curtailment of the practice of school inspection, and this policy has liberated a certain number of practitioners for service, direct or indirect, to the fighting line. To judge from some public announcements it would seem that there are parts of the country where the value of this arrangement is not appreciated. Thus the Aberdeen County Committee a few days ago appointed a medical inspector of school children at a salary of £500 per annum, and an assistant inspector at £300 per annum. Again, it is proposed in the counties of Roxburghshire and Selkirkshire to make the medical officer of health for the joint counties principal school medical officer at a salary not exceeding £1,000, and to provide him with an assistant at a salary of £400. These decisions and proposals are exciting some adverse comment in lay circles, and public opinion naturally inquires how they can be reconciled with the urgent demand of the War Office for more doctors. The general view appears to be that, without being completely suspended, the practice of school inspection might in the circumstances be considerably curtailed. Another point which has excited attention in the Border counties is the resolution of the local representatives of the British Medical Association that no medical man shall be allowed to accept the junior appointment at a salary of less than £500.

### THE SINKING OF THE "LUSITANIA."

While the destruction of the great passenger liner *Lusitania* does not differ in kind from that inflicted on other non-combatant vessels since the German so-called "blockade" commenced, it provides the most flagrant example of the murder of non-combatant and neutral persons since the war began. The motive appears to be to carry on the policy of "frightfulness" in the hope that the pressure of uninstructed public opinion may lead the Admiralty by altering their dispositions, and perhaps granting convoys, to provide other marks for the German submarine. That such a policy can be successful is unthinkable; but it is worth stating so that the public may be warned not to play into German hands by raising once more the senseless cry, "What is the Navy doing?" Institutional workers will have noted with interest that the most graphic story of the disaster, among the first batch of reports, was given to the *Times* correspondent by an American medical man, Dr. Moore, of South Dakota. Another survivor, Dr. C. E. Foss, of Montana, described to the same writer how he jumped overboard, and, after placing a floating oar within reach of two women in the water, propelled it and them towards a "canvas raft," on which they all clambered. Then he said he spent forty minutes in reviving by artificial respiration one of the women who had become unconscious. In reply to a question he stated that he heard there was a boat drill very early on Thursday morning, two days before the vessel was torpedoed. In any case, the loss of life has been, as it was intended to be, heavy. Its effect will be still further to stiffen the resolution of the nation to carry on the war to the bitter end—until, that is to say, the German military system is completely crushed.

### A BLOT ON NATIONAL INSURANCE ADMINISTRATION.

Since the introduction of the National Insurance Act there has been a notable change in the type of patients suffering from phthisis who have been admitted into the Lambeth Infirmary, resulting in a marked increase in the number of advanced cases, and a still more marked decrease in the early cases. With this change it has naturally become more difficult to persuade consumptive patients to come into and remain in the infirmary. Dr. Baly has made inquiries into the patients' reasons for refusing admission. These are, firstly, the unpleasant surroundings of the ward in which so many patients continuously die, and, secondly, the regularity of the diet, combined with the capricious appetite which is typical in patients suffering from this disease. He is taking steps to remove, as far as possible, the second cause of the complaint, although it is difficult to do much without overtaxing the staff. The first cause he found to be insurmountable under the conditions which existed in the infirmary. Very properly, under the circumstances, Dr. Baly has not felt justified in supporting the pressure which was brought to bear by outside authorities on these early cases who rightly refuse to enter the infirmary. Mr. Frank Briant, L.C.C., the chairman, states that the changes referred to by Dr. Baly are in a great measure due to the action of those administering the sanatoria benefits under the National Insurance Act, who send away as many as possible of those in the early stages of the disease. The facts contained in Dr. Baly's report exhibit with telling force the improper action of those administering sanatorium benefits under the National Insurance Act. Steps should be taken radically to change the present system by making provision in separate sanatoria for exclusively early cases. At present the lives of these unfortunate patients are being imperilled when they might be saved if placed under medical treatment with the essential hygienic precautions.

### "SECRET REMEDIES" AGAIN VINDICATED.

There was dismissed in the Court of Appeal last week an application for judgment or a new trial in an action tried before Mr. Justice Shearman in July 1914. The plaintiff was Mr. Stevens, managing director of Stevens & Co., Ltd., who claimed damages for alleged libel contained in the well-known publication of the British Medical Association called "Secret Remedies: What They Are and What They Contain." The passage complained of was to be found in Chapter III., headed "Consumption Cures," which, it was suggested, implied that a remedy sold by the plaintiff was a quack remedy of no medicinal value. The defendants denied that the words were capable of the alleged meaning, and that so far as the allegations consisted of fact they were true, and so far as of comment they were fair in a matter of public interest. In answer to the two questions put to the jury, (1) as to whether the words were libellous, and (2) whether the defendants had gone beyond fair criticism, the first was answered in the negative, and the second in the affirmative. A verdict for the defendants was returned. The plaintiff now applied for judgment or a new trial. The Court dismissed the application without calling on counsel for the respondents. In the course of his judgment Lord Justice Swinfen Eady said that the plaintiff had placed on the market what was represented in circulars as an absolute guarantee cure for consumption. The defendants' pamphlet was in substance a reprint of certain articles written by Mr. Harrison, which had appeared in the *British Medical Journal*. The defendants had not pleaded justification but only fair comment, and the plaintiff contended that an allegation of fraud went far beyond the limits of fair comment. As a matter of law it had been decided that where facts justified it words might convey a personal imputation and yet be fair comment. The matter was of grave public concern. The jury had all the facts before them, neither had there been any misdirection. The application failed and must be dismissed.

### ST. BARTHOLOMEW'S HOSPITAL AND PREVIOUS WARS.

The Rede Lecture, which was founded in the reign of Henry VIII. by Sir Robert Rede, Chief Justice of the Common Pleas, was delivered in the Senate House at Cambridge by Dr. Norman Moore, consulting physician to St. Bartholomew's Hospital. Taking as his subject, "St. Bartholomew's Hospital in Peace and War," the lecturer recalled its foundation in 1123 by Rahere, and noted that the first soldiers to come into relation with the hospital were the witnesses of a charter of Henry I., who afterwards took part in the wars of Stephen. The war at the end of King John's reign and during that of Henry III. between the French under Louis, eldest son of the French King, and the English under William the Marshal, was connected with the subsequent disturbances in London which ended in Constantine FitzAlulf's execution. In August 1222, not knowing he was to die the next day, FitzAlulf probably drew up the charter of benefaction to St. Bartholomew's, of which a copy has been preserved. Wat Tyler's mob in Smithfield was seen by the hospital staff a century later, and Wat himself, as he was dying, was pulled through the hospital gate. In the Great Rebellion, as during the present war, military dress was often seen at hospital committees and in the wards. A sister, added the lecturer, was reproved for being overheard to express the wish that Sir Thomas Fairfax's head might be placed on London Bridge. The first hospital steward after the Restoration had fought in defence of Basing House, and some wounded sailors in the second Dutch war were taken into the wards. Since that time till the last nine months the hospital had little to do with war, but it now provides beds for 200 wounded. This fortunate hiatus comes somewhat as a surprise till we remember how well the sea has served us "in the office of a wall," and how long since it is that English troops have been engaged in Flanders, the battleground of Western Europe, which modern methods of transit have brought almost to our doors.

### COMPULSORY NOTIFICATION OF MEASLES AND WHOOPING COUGH.

The Local Government Board has issued a circular-letter stating that the Board has had under consideration the measures which might be taken for controlling the spread of measles, German measles (rubeola), and whooping cough, and for reducing the mortality from these diseases. In the past the Board has approved Orders made by a number of sanitary authorities extending the provisions of the Infectious Disease (Notification) Act, 1889, to these diseases, but in several instances the authorities have subsequently rescinded the Order, as the system of notification under the Act, which requires notification of every case, has not proved to be of a value commensurate with the expense involved under the circumstances of administration hitherto adopted. Representations have been made to the Board in favour of a system under which the notification to the medical officer of health would be required of the first case in a household attended by a medical practitioner, and of each case in a household by the parents of the patients. The Board has further considered whether it should issue regulations applying this system generally throughout England and Wales. In the result the Board decided not to issue a general Order, but was willing to comply with an application from any sanitary authority for an Order making this system applicable to cases of the above diseases. It was provided that when a notification is received the medical officer of health shall make such inquiries and take such steps as are necessary or desirable to investigate the nature of each case and the source of infection, so as to prevent the spread of infection, and to remove all conditions favourable to infection. The Local Government Board proposes to include in the Order a clause empowering the sanitary authority to provide and contract for the supply of medical or nursing assistance for the poorer classes of the district who are suffering from any of these diseases.

### OBJECTIONS TO PARTIAL COMPULSION.

Dr. J. Priestley, medical officer of health for Lambeth, in a report to the Public Health Committee, states that he is of opinion that the compulsory notification of all cases of measles was not advisable at present, as the expense would be out of all proportion to the benefits likely to be gained, chiefly owing to the absence of sufficient hospital accommodation for the notified cases, the highly infectious nature of the disease in its early stages, and the age of the patients affected. He suggests that if partial notification of these diseases is to be adopted, the Board should itself issue a general Order to that effect, and not leave it to individual sanitary authorities to take the initiative, at least in so far as the Metropolis is concerned. He is of opinion that the proposal to insert a clause in the Order empowering an authority to provide medical or nursing assistance in these cases was both useful and valuable. Acting on the advice of Dr. Priestley, the Council have decided to acquaint the Board of Guardians with the views of their medical officer of health.

### THIS WEEK'S DRUG MARKET.

Business continues active and prices, generally speaking, are well maintained, although there have been none of those extraordinary advances which were not uncommon some little time ago. Synthetic drugs have now reached such high values that buying is limited to very small quantities, and with the possibility of further supplies becoming available buyers are naturally cautious. A good business has been done in tartaric acid, citric acid, and cream of tartar, and the tendency of prices is in an upward direction. Cod-liver oil has a lower tendency in price, and it is possible that prices may recede still further, the demand at the moment being quiet. The value of castor oil is not so firmly maintained. More plentiful supplies of ipecacuanha are now available. The official announcement that the Government propose to substitute for their taxing proposals a complete prohibition of the sale of spirits under three years of age disposes of the fear of a heavy increased taxation of spirituous medicines, but it is still possible that there will be a small extra tax on rectified spirits, which would involve at least some increase in the price of tinctures, although a small one compared with that which would have followed the first proposals of taxation. This point, however, is not made clear in the official announcement, and a further statement on the matter is awaited with interest.

### TO OUR READERS.

Contributions are specially invited from any of our readers to these columns. They should deal with topical subjects and news. They must be authenticated for the information of the Editor only. The minimum payment if published is 5s. There is no hard-and-fast rule as to space, but notes of about twenty lines in length are preferred.

